# *In planta* proximity dependent biotin identification (BioID)

**DOI:** 10.1038/s41598-018-27500-3

**Published:** 2018-06-15

**Authors:** Madiha Khan, Ji-Young Youn, Anne-Claude Gingras, Rajagopal Subramaniam, Darrell Desveaux

**Affiliations:** 1 Department of Cell and Systems Biology, University of Toronto, Toronto, Ontario M5S 3B2 Canada; 2 Ottawa Research and Development Centre, Agriculture and Agri-Food Canada, Ottawa, Ontario K1A 0C6 Canada; 3 Lunenfeld-Tanenbaum Research Institute, Sinai Health System, Toronto, Ontario M5G 1X5 Canada; 4 Department of Molecular Genetics, University of Toronto, Toronto, Ontario M5S 1A8 Canada; 5 Centre for the Analysis of Genome Evolution and Function, University of Toronto, Toronto, Ontario M5S 3B2 Canada

## Abstract

Proximity dependent biotin identification (BioID) has emerged as a powerful tool for studies of proteome architecture, including insoluble or membrane-associated proteins. The technique has been well established in mammalian cells but has yet to be applied to whole plant systems. Here we demonstrate the application of BioID on leaf tissues of the model plant *Arabidopsis thaliana*, thereby expanding the versatility of this important technique and providing a powerful proteomics tool for plant biologists.

## Introduction

Affinity purification coupled to mass spectrometry is a powerful approach to identify protein-protein interactions (PPIs) and understand the molecular dynamics of complex biological processes. There are however limitations to the type of interactions that are captured by this approach, since the interactors have to be both solubilized during cell lysis and maintain their association with one another throughout the affinity purification process. This is particularly problematic for associations that are less stable in solution, and for those that involve proteins that are difficult to solubilize in standard conditions, chief among those, membrane-associated complexes^1,2^. Proximity-dependent biotin identification (BioID) has emerged as a powerful tool for mapping proteome architecture of multiple biological processes, in addition to unraveling the proteome of highly insoluble compartments^1^. BioID uses a mutated version of the *Escherichia coli* biotin ligase, BirA*, to biotinylate proteins in a proximity-dependent manner^3,4^. The mutated biotin ligase readily releases reactive bioAMP, which covalently interacts with primary amines on proximal proteins. This method can be used to biotinylate proteins in proximity to a bait of interest that is translationally fused to BirA*. Once biotinylated, proteins can be extracted from cells under denaturing conditions, recovered by streptavidin affinity purification, and identified by mass spectrometry^4^. Studies in mammalian cells have demonstrated the effectiveness of BioID for physical mapping of functionally linked proteins to an array of cellular compartments^5,6^. In plants, the technique has only be applied in rice protoplasts to study proteins associated with OsFD2, a transcription factor involved in rice vegetative growth reported to form a complex with 14-3-3 proteins^7,8^. However, the BioID technique has yet to be applied to whole plant systems. The application of proteomic approaches in plants poses unique challenges relative to animal systems, including low cytoplasmic volume relative to cell wall mass, high protease and phosphatase content, and the predominance of ribulose-1,5-bisphosphate carboxylase/oxygenase (rubisco), which can interfere with protein detection and identification^2^. Despite these challenges, proteomics is an essential component of plant biology, and the application of the latest techniques is crucial for continued advances in the field^9^. Here we present the application of the BioID technique to the model plant *Arabidopsis thaliana* (hereafter Arabidopsis).

As a virulence strategy, bacterial pathogens such as *Pseudomonas syringae* translocate type III effector proteins into host cells via the type III secretion system. Many of these type III secreted effectors (T3Es) target proteins involved in plant immunity, thereby promoting bacterial growth^10^. Plants have evolved to recognize T3Es using resistance protein modules that contain nucleotide-binding and leucine-rich repeat (NLR) domain proteins, thereby inducing effector triggered immunity (ETI)^11,12^.

HopF2b^*Pt0*DC3000^ (hereafter HopF2) is a membrane-targeted, type III secreted effector that promotes *Pseudomonas syringae* growth in Arabidopsis^13,14^. HopF2 regulates the activity of the membrane localized immunity regulator RIN4, preventing its degradation by another T3E effector, AvrRpt2, ultimately suppressing AvrRpt2-induced ETI^12,14–16^. In a previous proteomic analysis of HopF2 using affinity purification coupled to mass spectrometry (AP-MS), we identified several membrane-associated proteins, consistent with the predicted myristoylation of HopF2 and its membrane localization *in planta*^14,17–19^. We therefore used HopF2 tagged BirA* and BirA* alone as proof of concept to develop BioID in Arabidopsis and to compare the proximal proteome of membrane-associated and soluble baits.

## Results and Discussion

**Figure 1:**
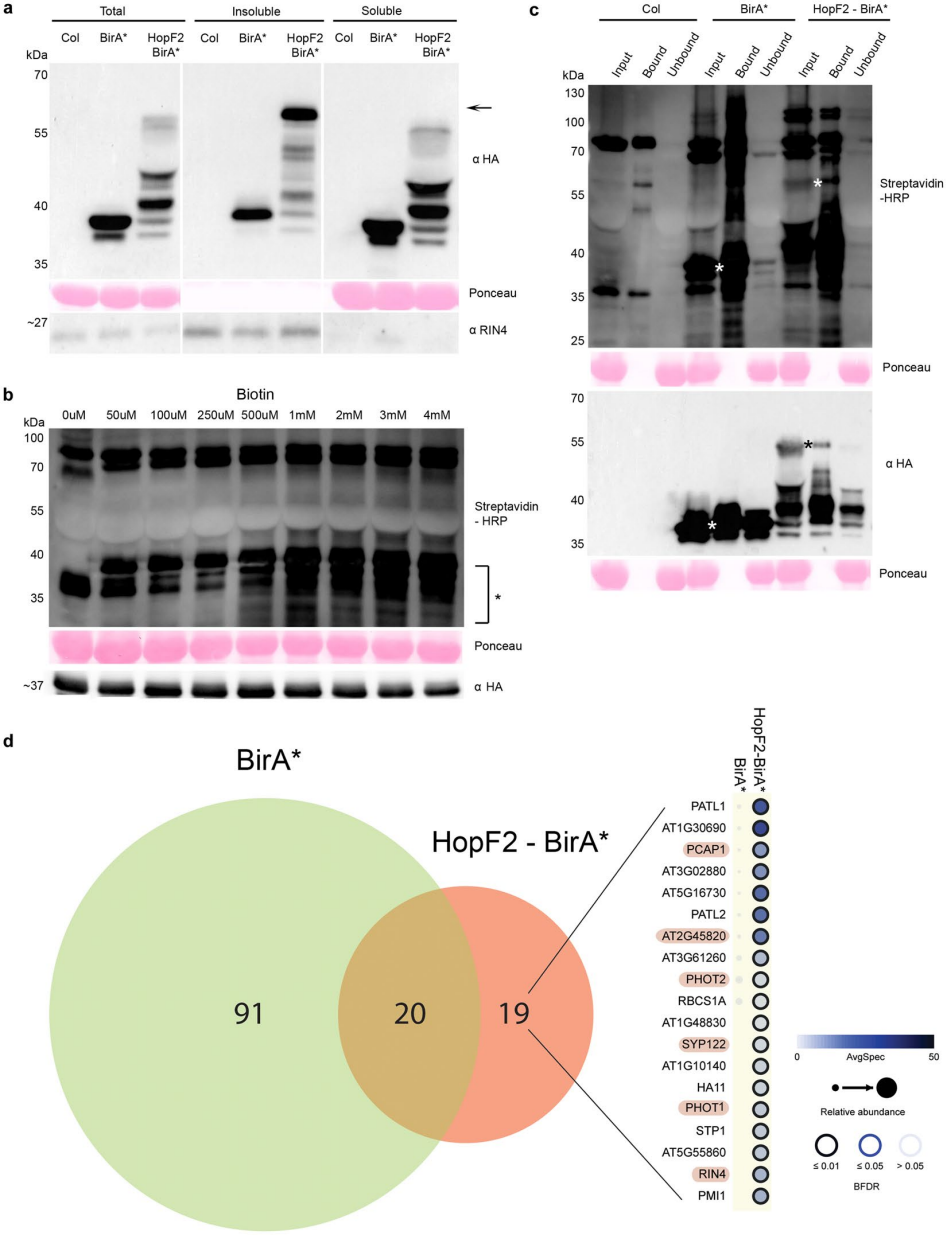
Proximity dependent biotin identification (BioID) in Arabidopsis. (**a**) Immunoblot of total, insoluble (membrane) and soluble extracts from Col-0 and transgenic plants expressing BirA* (~35 kDa) or HopF2-BirA* (~60 kDa; marked by an arrow) using HA antibody. Extracts were probed with RIN4 antibody as a fractionation control. Ponceau staining is presented as a loading control. Since RuBisCO was not found in the insoluble fraction, RIN4 also serves as a loading control for insoluble fraction. (Note that proteins from the insoluble samples migrated slower on the SDS-PAGE giving them an apparent higher molecular weight, which may be due to differences in sample buffer composition after resuspension of the insoluble pellet (see Methods)). (Note that the images were cropped to show relevant samples and uncropped images are available as Supplementary Information). **(b)** Immunoblot showing biotin labeling efficiency across concentration gradient (0 μM–4 mM) of exogenous biotin applied to BirA* plants. Biotinylated proteins from total extracts were detected using HRP-conjugated streptavidin. Saturation is most obvious in lower molecular bands (<35 kDa) indicated with an asterisk. HA antibody was used to detect baits in all extracts. (Note that the images were cropped to show relevant samples and uncropped images are available as Supplementary Information). **(c)** Streptavidin affinity purification of total leaf extracts from Col-0 (parental ecotype), and transgenic plants stably expressing BirA* or HopF2-BirA*, infiltrated with 2 mM exogenous biotin (see Methods). Total protein extract (Input), biotinylated proteins pulled down by streptavidin affinity purification (Bound) and proteins not retained by affinity purification (Unbound) were loaded from each sample. Biotinylated proteins were visualized with HRP-conjugated streptavidin (top panel) and bait expression monitored using HA antibody (bottom panel). Expected bait proteins are marked by asterisks* on the Streptavidin-HRP blot and HA blot. (Note that the images were cropped to show relevant samples and uncropped images are available as Supplementary Information). **(d)** Venn Diagram showing the number of significant preys identified in BirA* or HopF2-BirA* across two independent biological replicates relative to three biological replicates of Col-0 (BFDR ≤ 0.01). Preys unique to HopF2-BirA* bait are visualized on the right side as a dot plot, which shows list of preys on the y-axis and its interacting baits on top^22,23^. Simply, the color of the dot represents the average spectral counts of identified prey, the size of the dot reflects the relative abundance of a prey across two baits, and the edge colour indicates the BFDR value associated with each bait-prey interaction (see legend). Preys known to be involved in plant immunity are highlighted in pink.

We generated transgenic Arabidopsis plants that conditionally express HA epitope tagged HopF2-BirA*^17^ (~60 kDa; “membrane”) and BirA* (~35 kDa; “soluble”) under the control of a dexamethasone-inducible promoter. Consistent with previous findings^17,18^, BirA*-tagged, full-length HopF2 localized predominantly in the insoluble (crude membrane) fraction, with fragments of HopF2-BirA* present in the soluble fraction (see Methods for extraction conditions; Fig. 1a). The bands observed in the HopF2-BirA* soluble fraction most likely lack the myristoylation sequence required for membrane localization^17^. BirA* was localized mainly to the soluble fraction, however, a limited amount of the protein also partitioned to the insoluble fraction potentially a result of some BirA* membrane association or localization to nuclei due to its DNA-binding domain^20^. The membrane protein RIN4 was used to confirm the enrichment of membrane proteins in the insoluble fraction. As expected, RIN4 was detected in the total extract and in the insoluble fraction, but not significantly in the soluble fraction^21^ (Fig. 1a).

In mammalian cells, 50 μM of exogenously applied biotin is sufficient for BirA*-mediated biotinylation^4^. To establish the level of exogenous biotin required for efficient labeling *in planta*, we tested biotinylation efficiency in BirA* transgenic plants using a biotin gradient up to 4 mM. We infiltrated biotin directly into leaf tissues, and harvested tissues 22 hrs post infiltration (see Methods for details). We detected biotinylation of proteins in total leaf extracts with streptavidin conjugated horseradish peroxidase (HRP) (see Methods). The saturation point for biotinylation by BirA* in Arabidopsis was reached with approximately 1 mM biotin (Fig. 1b). Immunoblot with HA antibodies was used to confirm comparable BirA* expression in all extracts (Fig. 1b). To ensure efficient biotinylation, we applied 2 mM exogenous biotin in all the subsequent experiments. We also optimized the timing of protein expression with biotin infiltration and determined that optimal protein expression and biotinylation occurred 24 hours following co-application of dexamethasone and 2 mM biotin (Supplementary Fig. 1).

Following optimization of the exogenous biotin concentration, we used streptavidin affinity purification to pull down biotinylated proteins from total extracts of untransformed Arabidopsis (Col-0), transgenic BirA* and HopF2-BirA* plants. Protein extracts from BirA* and HopF2-BirA* lines had extensive amounts of biotinylated proteins relative to Col-0 extracts (Fig. 1c). A majority of the biotinylated proteins were pulled down by streptavidin affinity capture, as observed by the depletion of biotinylated proteins from the unbound fraction (Fig. 1c). These results demonstrate that BirA* tagged proteins can effectively biotinylate proteins *in vivo* in Arabidopsis and that these proteins can be effectively purified from Arabidopsis extracts by streptavidin-conjugated resin.

Next, streptavidin affinity purified proteins were identified by LC-MS/MS (see details in Methods). Two biological replicate purifications of each BirA* and HopF2-BirA* and three biological replicates of the untransformed Arabidopsis (Col-0) samples were processed independently. Over 500 proteins were identified in each sample with a protein identification iProphet score ≥0.95 (Supplementary Dataset 1). We utilized Significance Analysis of INTeractome (SAINT) to identify proteins significantly labeled by soluble BirA* or the insoluble HopF2-BirA* relative to the Col-0 negative control^22,23^ (Supplementary Dataset 2, Supplementary Fig. 2). SAINT calculates, for each prey protein identified in a purification, the probability of true interaction based on the quantitative recovery of a prey in a purification of a bait, across the detection of the same preys in the negative control runs (Col-0), and reports as high confidence those preys reproducibly detected across both biological replicates^22,23^. SAINT analysis identified 39 HopF2-BirA* preys and 111 BirA* prey proteins, respectively (Bayesian FDR ≤ 0.01, Fig. 1d, Supplementary Fig. 2, Supplementary Dataset 2). Among these prey proteins, 19 were unique to the HopF2-BirA* bait (Table 1), whereas 91 were unique to BirA* (Supplementary Table 1), and 20 were common to both baits (Supplementary Table 2), at the confidence threshold used (BFDR ≤ 0.01). We analysed prey proteins uniquely associated with each bait for localization as well as their presence in our previously published HopF2 AP-MS proteomic dataset^18^ (Table 1, Supplementary Table 1 and Supplementary Table 2). 95% (18/19) of proteins uniquely associated with HopF2-BirA* localized to membranes, whereas only 43% (39/91) of BirA* prey proteins localized to membranes, based on Universal Protein Resource (UniProt) (Table 1 and Supplementary Table 1), showing a statistically significant increase in the proportion of membrane proteins in HopF2-BirA* preys (Fisher exact test p < 0.001). 58% (11/19) of prey proteins uniquely associated with HopF2-BirA*, were present in the previously published AP-MS dataset, whereas less than 7% (6/91) of preys unique to BirA* were present in the AP-MS dataset^18^ (Table 1**;** Supplementary Table 1).

**Table 1:** Significant Preys identified in HopF2-BirA* but not in BirA*.

Preys	Common Name	Localization^a,b^	Identified by AP-MS^c,d^
At1g30690	PATL4	Membrane	
At1g72150	PATL1	Membrane	Yes (gi|15028181)
At5g16730		Non-membrane	
At1g22530	PATL2	Membrane	Yes (gi|6587836)
At2g45820^◆^	REM1.3	Membrane	Yes (gi|601843)
At3g02880		Membrane	Yes (gi|10176968)
At3g61260	REM1.2	Membrane	Yes (gi|6850893)
At1g11260	STP1	Membrane	Yes (gi|5734730)
At3g25070^◆^	RIN4	Membrane	Yes^e^
At1g10140		Membrane	
At1g42550	PMI1	Membrane	
At5g55860		Membrane	
At3g45780^◆^	PHOT1	Membrane	
At3g52400^◆^	SYP122	Membrane	
At5g62670	HA11	Membrane	Yes (gi|8809663)
At1g48830	RPS7A	Membrane	
At4g20260^◆^	PCAP1	Membrane	Yes (gi|1550738)
At1g67090	RBCS1A	Membrane	Yes (gi|11762170)
At5g58140^◆^	PHOT2	Membrane	Yes (gi|10176790)

^a^Membrane or non-membrane based on subcellular localization information on Uniprot.org.

^b^94.74% of HopF2-BirA*-specific preys are membrane localized.

^c^Identified in the AP-MS data of Hurley *et al*.^18^, in at least one HopF2 replicate and not in the negative control^18^.

^d^57.89% of HopF2-BirA*-specific preys were also identified by AP-MS^18^.

^e^Detected by western blotting^14,18^.

^◆^Proteins previously identified as being involved in plant immunity^21,24–27^.

HopF2-BirA* BioID identified immunity related proteins that had been previously identified by AP-MS including RIN4^21^, REMORIN1.3 (REM1.3, At2g45820)^24^, PLASMA-MEMBRANE ASSOCIATED CATION-BINDING PROTEIN 1 (PCAP1)^25,26^, and PHOTOTROPIN 2 (PHOT2)^27^ (Fig. 1d, Table 1)^18^. Interestingly, RIN4, a direct target of HopF2 and a major plant immunity regulator identified in this report was only detected by Western analysis in the AP-MS study^18^, underscoring the potential capacity of the BioID technique. In addition to known targets of HopF2, BioID also identified new HopF2-associated proteins including immunity-related protein SYNTAXIN OF PLANTS 122 (SYP122)^28–30^ and PHOTOTROPIN 1 (PHOT1)^24^, as well as plastid related proteins PMI1 and At5g16730, and other uncharacterized proteins At1g10140 and At5g55860. We speculate that some of these proteins may be involved in processes targeted by HopF2 to promote pathogenesis. On the other hand, not all the known HopF2 interactors were identified in our BioID screen including MAP KINASE KINASE5 (MKK5)^31^ and BRASSINOSTEROID INSENSITIVE 1-associated receptor kinase 1(BAK1)^19^. This could be due to the limited range of BirA* biotinylation (~10 nm radius) or due to a lack of accessible primary amines on proximal proteins for biotinylation^32,33^. Nevertheless, our BioID results identified common but also unique sets of protein interactions compared to AP-MS^18^, which is in line with a comparison of BioID and FLAG AP-MS datasets using histones H2B and H3 as baits in mammalian cells^18,34^ (Fig. 1d). Future experiments will be required to establish which preys represent HopF2-BirA* specific targets. These will include the use of a general membrane-localized BirA* control as well as independent protein interaction validation methods such as bimolecular fluorescence complementation (BiFC) or co-immunoprecipitation (CoIP) techniques. Overall our results demonstrate that BioID can be used to identify biologically-relevant proximal proteins of BirA*-fused baits *in planta* and can distinguish proximal proteomes associated with different bait locations.

In conclusion, we have demonstrated that BioID can be applied to leaf tissues of the model plant Arabidopsis. This technique will be a strong addition to the proteomic toolbox of plant biologists, particularly well-suited for the study of membrane associated proteins. *In planta* BioID will provide a powerful approach to probe plant proteome organization, including subcellular compartments and macromolecular structures, all in physiologically relevant contexts.

## Methods

### Plant growth conditions

*Arabidopsis thaliana* plants were grown with 9 h light (~130 microeinsteins m^−2^ s^−1^) and 15 h darkness at 22 °C in Sungro soil supplemented with 20:20:20 fertilizer. Col-0 wild-type was used as negative control and as background for all transgenic lines.

### Cloning

Phusion polymerase (New England Biolabs) was used for all cloning and constructs were confirmed by sequencing. We created C-terminally tagged HopF2 to allow the myristoylation site to be accessible (HopF2-BirA*-FLAG-HA, referred as HopF2-BirA*). HopF2 and BirA*-FLAG fragments were amplified from previously described pBBR1 MCS-2 and pDEST5-BirA*-FLAG-pcDNA5-FRT-TO respectively^14,35^. These fragments were ligated using overlap extension PCR^36^. Ligated products were cloned into pDB vector at the StuI restriction site with an ATG start codon and in-frame with the C-terminal hemagglutinin (HA) tag using In Fusion Cloning kit^37^ (Takara Clontech:638909). BirA*-FLAG-HA (referred as BirA*) was also cloned into the pDB vector at the StuI restriction site using In Fusion Cloning kit (Takara Clontech:638909).

### Transgenic lines

Wildtype *Arabidopsis thaliana* (Col-0) plants were transformed with pBD::BirA*-FLAG-HA or pBD::HopF2*-BirA*-FLAG-HA using the floral dip method^38^ with the *Agrobacterium* strain GV3101. Transgenic plants were selected by Basta resistance and confirmed by protein expression from individual plants using Western method as previously described^39^. Homozygosity of T3 lines were determined by their segregation ratios on Murashige and Skoog media and 6 mg/L bialaphos.

### Bait protein solubility test

For lysate fractionation, 10 leaves from different plants were detached and floated on 30 μM dexamethasone (DEX) 22hrs on a shaker at 25 °C, then frozen in liquid nitrogen. The leaf tissue was ground in extraction buffer containing 20 mM Tris (pH 8.0), 100 mM NaCl and 1 mM DTT. The crude extract was cleared by centrifugation at 5000 g for 10 minutes at 4 °C. An aliquot of the supernatant was saved as total extract (120 μg of protein). Supernatant was centrifuged again at 20000 g for 30 minutes at 4 °C. Supernatant from this spin was used as soluble fraction. The insoluble pellet was resuspended in extraction buffer with addition of 1% Triton X-100 and 0.4% sodium dodecyl sulfate (SDS), vortexed for 15 minutes and cleared by centrifugation at 20000 g for 30 minutes. Laemmli SDS-PAGE loading buffer was added to the tube and boiled for 5 minutes. 18 μg of protein was separated on 10% SDS-PAGE gels, blotted onto nitrocellulose membranes and detected using HA antibodies (Roche no. 11867423001) or RIN4 antibodies^14^.

For biotin concentration gradients, 10 leaves from 4–5 week old transgenic BirA* plants were infiltrated with various concentrations of biotin by hand using a needleless syringe and floated overnight (22hrs) in 30 μM DEX on a shaker at 25 °C. After incubation, the leaves were frozen and protein extraction was performed as described^39^. SDS-PAGE gels were run as mentioned above. Streptavidin-horseradish peroxidase conjugate (GE Healthcare no. RPN1231) or HA antibodies (Roche no. 11867423001) were used at 1:2000 and 1:15000 dilution respectively.

For time course treatment of HopF2-BirA* with 30 μM DEX, plants were infiltrated with 2 mM Biotin and then floated on DEX solution for 0, 8, 16, 24 or 32 hrs on a shaker at 25 °C. After incubation, the leaves were frozen and protein extraction was performed as described^39^. SDS-PAGE gels were run as mentioned above. Streptavidin-HRP (GE Healthcare no. RPN1231) or HA antibodies (Roche no. 11867423001) were used to detect proteins.

### BioID Sample preparation for MS

For streptavidin-based affinity purification, 5 g of leaf tissue from 4–5 week old Columbia wildtype, BirA* and HopF2-BirA* transgenic plants was infiltrated with 2 mM biotin, floated in 30 μM DEX 24hrs on a shaker at 25 °C, then frozen in liquid nitrogen. The tissue was ground using extraction protocol described above for protein localization with the addition of 1% of plant protease inhibitor cocktail (Sigma no. P9599) to the extraction buffer (all buffers and wash solutions were made with MS-grade H_2_O). The insoluble and soluble fractions were combined before affinity purifications. The combined fractions were run through Amicon Ultra-4 centrifugal filter units with Ultracel-10 membrane (EMD Millipore no. UFC8010) and washed four times with 4 mL of extraction buffer without detergents to remove excess biotin in lysates. Concentrated 1 mL of supernatant (12 mg of protein) with additional 1 mM DTT and 1% protease inhibitor cocktail (Sigma no. P9599) was used for affinity purification with 30 μL of prewashed 50% slurry mix of streptavidin sepharose beads (GE healthcare no. 17511301) overnight in a rotator at 4 °C. After affinity purification, supernatant with leftover proteins was used as unbound fraction. Streptavidin beads were washed once in extraction buffer without detergents (1 mL per wash), once in SDS buffer (2% SDS, 50 mM Tris pH 7.5) two more times in extraction buffer (1 mL per wash) and three times in 50 mM ammonium bicarbonate (ABC; pH 8.0). An aliquot of the beads was saved for western blots as the bound fraction. Note that all buffers used for affinity purification and on bead digest are made in HPLC grade water.

### On bead digestion

After affinity purification and removal of all washing buffer, beads were resuspended in 200 μL of 50 mM ABC (pH 8.0) with 1 μg of trypsin (Sigma no. T6567) added and incubated at 37 °C with agitation for four hours after which an additional 1 μg is added and samples left agitating overnight at 37 °C. Beads are pelleted (500 g, 2 min) and the supernatant was transferred to a fresh 1.5 mL tube. The beads were rinsed with 100 μL of MS-grade H_2_O and the rinses were combined with original supernatant. The pooled fraction was acidified to 2% formic acid and centrifuged at 16000 g for 5 minutes. The top 80% supernatant volume was carefully transferred to a new 1.5 mL tube to remove any residual streptavidin resin.

### MS data acquisition

Affinity-purified digested material from 5 g of leaf tissues was resuspended in 6 μL of 5% formic acid, and 5 μL injected by autosampler onto a spray tip formed from a fused silica capillary column (0.75 μm ID, 350 μm OD) generated using a laser puller. The 10 to 12 cm column was packed with C18 reversed-phase material (Reprosil-Pur 120 C18-AQ, 3 μm) by pressure bomb loading in MeOH and was pre-equilibrated with buffer A (100% H_2_O, 0.1% formic acid). The column was placed in-line with a LTQ-Orbitrap Elite (Thermo Fisher Scientific) equipped with a nanoelectrospray ion source (Proxeon, Thermo Fisher Scientific) connected in-line to a NanoLC-Ultra 2D plus HPLC system (Eksigent, Dublin, USA). The LTQ-Orbitrap Elite instrument under Xcalibur 2.0 was operated in the data dependent mode to automatically switch between MS and up to 10 subsequent MS/MS acquisition. The HPLC fractionation was performed on a acetonitrile gradient over 125 min. For the first twenty min, the flow rate was of 400 μL/min in 2% buffer B (100% Acetonitrile, 0.1% formic acid). The flow rate was then reduced to 200 μl/minwith a linear gradient up to 35% buffer B till 95.5 min. Buffer B was then increased to 80% over 5 min and maintained at that level until 107 min. The mobile phase was then reduced 2% buffer B until the end of the run (125 min).

### MS/MS data analysis

All Thermo RAW files were saved in our local interaction proteomics LIMS, ProHits^40^. mzXML files were generated from ThermoFinnigan RAW files using the ProteoWizard^41^ converter, implemented within ProHits (–filter “peakPicking true2”–filter “msLevel2”). The searched database contained the *Arabidopsis thaliana* RefSeq protein database (version 77; taxonomy ID:3702) supplemented with and *Pseudomonas syringae* proteins (HopZ1a Refseq: DQ986440.1 and HopZ1A C216A catalytic mutant), “common contaminants” from the Max Planck Institute (http://141.61.102.106:8080/share.cgi?ssid=0f2gfuB), and the Global Proteome Machine (GPM; http://www.thegpm.org/crap/index.html) and common sequence tags (BirA_R118G and Streptavidin). In total, 71480 sequences (forward and reverse) were searched by Mascot and Comet, allowing for trypsin specificity and two missed cleavage sites. Methionine oxidation and asparagine/glutamine deamidation were set as variable modifications. The fragment mass tolerance was 0.6 Da and the mass window for the precursor was ±12 ppm. The resulting Comet and Mascot search results were individually processed by PeptideProphet^42^, and peptides were assembled into proteins using parsimony rules first described in ProteinProphet^43^ into a final iProphet^43^ protein output using the Trans-Proteomic Pipeline (TPP; Linux version, v0.0 Development trunk rev 0, Build 201303061711). TPP options were as follows: for the Elite Orbitrap files, general options are -p0.05 -x20 -PPM - d“DECOY”, iProphet options are pPRIME and PeptideProphet options are pPAEd. All proteins with a minimal iProphet protein probability of 0.05 were parsed to the relational module of ProHits. Note that for analysis with SAINT, only proteins with iProphet protein probability ≥0.95 are considered. This corresponds to an estimated protein level FDR of ~0.5%. This data set consisting of 7 raw files have been deposited in MassIVE (http://massive.ucsd.edu) with accession number MSV000080913.

### Significant Analysis of INTeractome (SAINT)

SAINTexpress analysis was performed using version exp3.3 with two biological replicates for each HopF2-BirA* and BirA* baits^23^. Bait protein samples were analyzed alongside 3 negative control runs, consisting of purifications from Col-0 plants also infiltrated with 2 mM Biotin^23^. Each biological replicate was considered separately for the initial scoring (the SAINT probability score is scaled from 0 to 1, with 1 being the highest). The scores were next averaged (AvgP), and a Bayesian False Discovery Rate (BFDR) estimation was then applied to the entire AvgP score distribution. Interactions with a calculated BFDR ≤0.01 were considered high confidence. Supplementary Dataset 2 shows the entire SAINT analysis with the exception of several preys that was manually removed: Streptavidin, trypsin, and ENSEMBL:ENSBTAP00000016242 (a tubulin protein from Bovine). Visualization was performed with the dotplot tool at ProHits-viz.lunenfeld.ca^44^ with default options; once any protein passes the 0.01 BFDR threshold in any bait-prey pair, all its quantitative values across all baits are recovered, irrespective of the individual BFDR of the given bait-prey pair.

### Comparison of membrane localization

Prey proteins identified by SAINT analysis for BirA* and HopF2-BirA* were searched on the UniProt database for cellular localization. All proteins present in any published membrane dataset were identified as ‘membrane localized’ (Table 1, Supplementary Table 1 and 2). For statistical comparison, we used a Fisher exact test to compare the proportion of membrane protein in soluble BirA* versus insoluble HopF2-BirA*.

Prey proteins were also checked for presence in the HopF2 AP-MS dataset^18^. Proteins with peptide count >2 identified in any DEX treated experiment and not present in the control experiment were included as being present in the HopF2 dataset (Table 1, Supplementary Table 1 and 2)^18^.

## Electronic supplementary material

Supplementary Information

Dataset 1

Dataset 2

## References

[1] Kim DI, Roux KJ (2016). Filling the Void: Proximity-Based Labeling of Proteins in Living Cells. Trends Cell Biol..

[2] Hurley B, Subramaniam R, Guttman DS, Desveaux D (2014). Proteomics of effector-triggered immunity (ETI) in plants. Virulence.

[3] Choi-Rhee E, Schulman H, Cronan JE (2004). Promiscuous protein biotinylation by Escherichia coli biotin protein ligase. Protein Sci..

[4] Roux KJ, Kim DI, Raida M, Burke B (2012). A promiscuous biotin ligase fusion protein identifies proximal and interacting proteins in mammalian cells. J. Cell Biol..

[5] Gupta GD (2015). A Dynamic Protein Interaction Landscape of the Human Centrosome-Cilium Interface. Cell.

[6] Youn JY (2018). High-Density Proximity Mapping Reveals the Subcellular Organization of mRNA-Associated Granules and Bodies. Mol. Cell.

[7] Lin Q (2017). Screening of Proximal and Interacting Proteins in Rice Protoplasts by Proximity-Dependent Biotinylation. Front. Plant Sci..

[8] Tsuji H, Nakamura H, Taoka KI, Shimamoto K (2013). Functional diversification of FD transcription factors in rice, components of florigen activation complexes. Plant Cell Physiol..

[9] Heazlewood JL (2011). The green proteome: challenges in plant proteomics. Front. Plant Sci.

[10] Block A, Alfano JR (2011). Plant targets for Pseudomonas syringae type III effectors: virulence targets or guarded decoys?. Curr. Opin. Microbiol..

[11] Jones JDG, Vance RE, Dangl JL (2016). Intracellular innate immune surveillance devices in plants and animals. Science (80-.)..

[12] Khan M, Subramaniam R, Desveaux D (2016). Of guards, decoys, baits and traps: Pathogen perception in plants by type III effector sensors. Curr. Opin. Microbiol..

[13] Lo T, Koulena N, Seto D, Guttman DS, Desveaux D (2016). The HopF family of *Pseudomonas syringae* type III secreted effectors. Mol. Plant Pathol..

[14] Wilton M (2010). The type III effector HopF2pto targets Arabidopsis RIN4 protein to promote Pseudomonas syringae virulence. Proc Natl Acad Sci USA.

[15] Axtell MJ, Staskawicz BJ (2003). Initiation of RPS2-specified disease resistance in Arabidopsis is coupled to the AvrRpt2-directed elimination of RIN4. Cell.

[16] Mackey D (2003). Arabidopsis RIN4 is a target of the type III virulence effector AvrRpt2 and modulates RPS2-mediated resistance. Cell.

[17] Robert-Seilaniantz A, Shan L, Zhou JM, Tang X (2006). The Pseudomonas syringae pv. tomato DC3000 type III effector HopF2 has a putative myristoylation site required for its avirulence and virulence functions. Mol Plant Microbe Interact.

[18] Hurley B (2014). The Pseudomonas syringae type III effector HopF2 suppresses arabidopsis stomatal immunity. PLoS One.

[19] Zhou J (2014). The Pseudomonas syringae effector HopF2 suppresses Arabidopsis immunity by targeting BAK1. Plant J..

[20] Kim DI (2016). An improved smaller biotin ligase for BioID proximity labeling. Mol Biol Cell.

[21] Mackey D (2002). RIN4 interacts with Pseudomonas syringae Type III effector molecules and is required for RPM1-mediated resistance in Arabidopsis. Cell.

[22] Choi H (2011). SAINT: probabilistic scoring of affinity purification-mass spectrometry data. Nat. Methods.

[23] Teo G (2014). SAINTexpress: improvements and additional features in Significance Analysis of Interactome software. J Proteomics.

[24] Bozkurt TO (2014). The Plant Membrane-Associated REMORIN1.3 Accumulates in Discrete Perihaustorial Domains and Enhances Susceptibility to Phytophthora infestans. Plant Physiol..

[25] Ide Y (2007). Molecular properties of a novel, hydrophilic cation-binding protein associated with the plasma membrane. J. Exp. Bot..

[26] Vijayapalani P, Maeshima M, Nagasaki-Takekuchi N, Miller WA (2012). Interaction of the trans-frame potyvirus protein P3N-PIPO with host protein PCaP1 facilitates potyvirus movement. PLoS Pathog..

[27] Jeong RD (2010). Cryptochrome 2 and phototropin 2 regulate resistance protein-mediated viral defense by negatively regulating an E3 ubiquitin ligase. Proc Natl Acad Sci USA.

[28] Nühse TS, Boller T, Peck SC (2003). A Plasma Membrane Syntaxin Is Phosphorylated in Response to the Bacterial Elicitor Flagellin. J. Biol. Chem..

[29] Assaad FF (2004). G Protein-coupled Receptor Kinase 2–mediated Phosphorylation of Ezrin Is Required for G Protein- coupled Receptor–dependent Reorganization of the Actin Cytoskeleton. Mol. Biol. Cell.

[30] Zhang Z (2007). A SNARE-protein has opposing functions in penetration resistance and defence signalling pathways. Plant J..

[31] Wang Y (2010). A Pseudomonas syringae ADP-ribosyltransferase inhibits Arabidopsis mitogen-activated protein kinase kinases. Plant Cell.

[32] Kim DI (2014). Probing nuclear pore complex architecture with proximity-dependent biotinylation. Proc Natl Acad Sci USA.

[33] Roux KJ (2013). Marked by association: Techniques for proximity-dependent labeling of proteins in eukaryotic cells. Cell. Mol. Life Sci..

[34] Lambert J (2015). Proximity biotinylation and affinity purification are complementary approaches for the interactome mapping of chromatin-associated protein complexes. J Proteomics.

[35] Couzens AL (2013). Protein interaction network of the mammalian Hippo pathway reveals mechanisms of kinase-phosphatase interactions. Sci. Signal..

[36] Heckman KL, Pease LR (2007). Gene splicing and mutagenesis by PCR-driven overlap extension. Nat Protoc.

[37] Lewis JD (2013). The Arabidopsis ZED1 pseudokinase is required for ZAR1-mediated immunity induced by the Pseudomonas syringae type III effector HopZ1a. Proc. Natl. Acad. Sci. USA.

[38] Clough SJ (1998). & Bent, a F. Floral dip: a simplified method for Agrobacterium-mediated transformation of Arabidopsis thaliana. Plant J..

[39] Lewis JD, Wu R, Guttman DS, Desveaux D (2010). Allele-specific virulence attenuation of the Pseudomonas syringae HopZ1a type III effector via the Arabidopsis ZAR1 resistance protein. PLoS Genet..

[40] Liu G (2010). ProHits: an integrated software platform for mass spectrometry- based interaction proteomics. Nat. Biotechnol..

[41] Adusumilli R, Mallick P (2017). Data conversion with ProteoWizard msConvert. Methods Mol Biol.

[42] Keller A, Nesvizhskii AI, Kolker E, Aebersold R (2002). Empirical statistical model to estimate the accuracy of peptide identifications made by MS/MS and database search. Anal. Chem..

[43] Nesvizhskii AI, Keller A, Kolker E, Aebersold R (2003). A statistical model for identifying proteins by tandem mass spectrometry. Anal. Chem..

[44] Knight, J. D. R. *et al*. ProHits-viz: a suite of web-tools for visualizing interaction proteomics data. **14**, 645–646 (2017). 10.1038/nmeth.4330 PMC5831326 28661499

