# Supplementary material for: In planta proximity dependent biotin identification (BioID)
Source: Sci Rep. 2018 Jun 15;8:9212. doi: 10.1038/s41598-018-27500-3 (PMC6004002; doi:10.1038/s41598-018-27500-3)
Supplement: Supplementary file 1 — Supplementary Information [file 41598_2018_27500_MOESM1_ESM.pdf]

***In planta* proximity dependent biotin identification (BioID)**

Madiha Khan*[1, 2], Ji-Young Youn*[3], Anne-Claude Gingras[3,4], Rajagopal Subramaniam[2] and Darrell Desveaux[1,5]

*Authors contributed equally to this paper

[1]Department of Cell and Systems Biology, University of Toronto, Toronto, Ontario M5S 3B2, Canada

[2]Ottawa Research and Development Centre, Agriculture and Agri-Food Canada, Ottawa, Ontario K1A 0C6, Canada

[3]Lunenfeld-Tanenbaum Research Institute, Sinai Health System, Toronto, Ontario M5G 1X5, Canada

[4]Department of Molecular Genetics, University of Toronto, Toronto, Ontario M5S 1A8, Canada

[5]Centre for the Analysis of Genome Evolution and Function, University of Toronto, Toronto, Ontario M5S 3B2, Canada

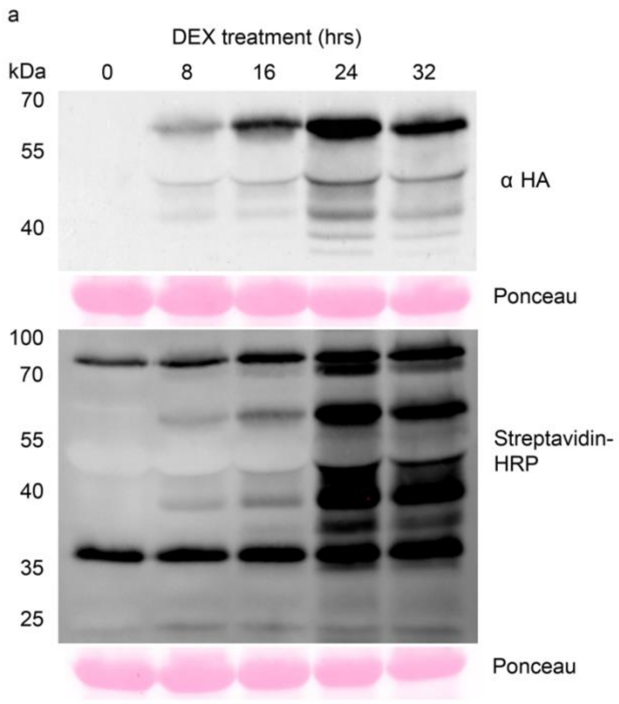

Supplementary Figure 1: Time course treatment of HopF2-BirA* with DEX

Total protein extract from HopF2-BirA* (60kDa) floated in 30μM DEX for 0, 8, 16, 24, 32 hrs was probed with HA-antibody (top panel) or streptavidin-HRP (bottom panel). Highest HopF2-BirA* expression and biotin labeling was observed following 24hrs treatment. Ponceau staining is presented as a loading control. (Note that the images were cropped to show relevant samples and uncropped images are available in Supplementary Figure 3).

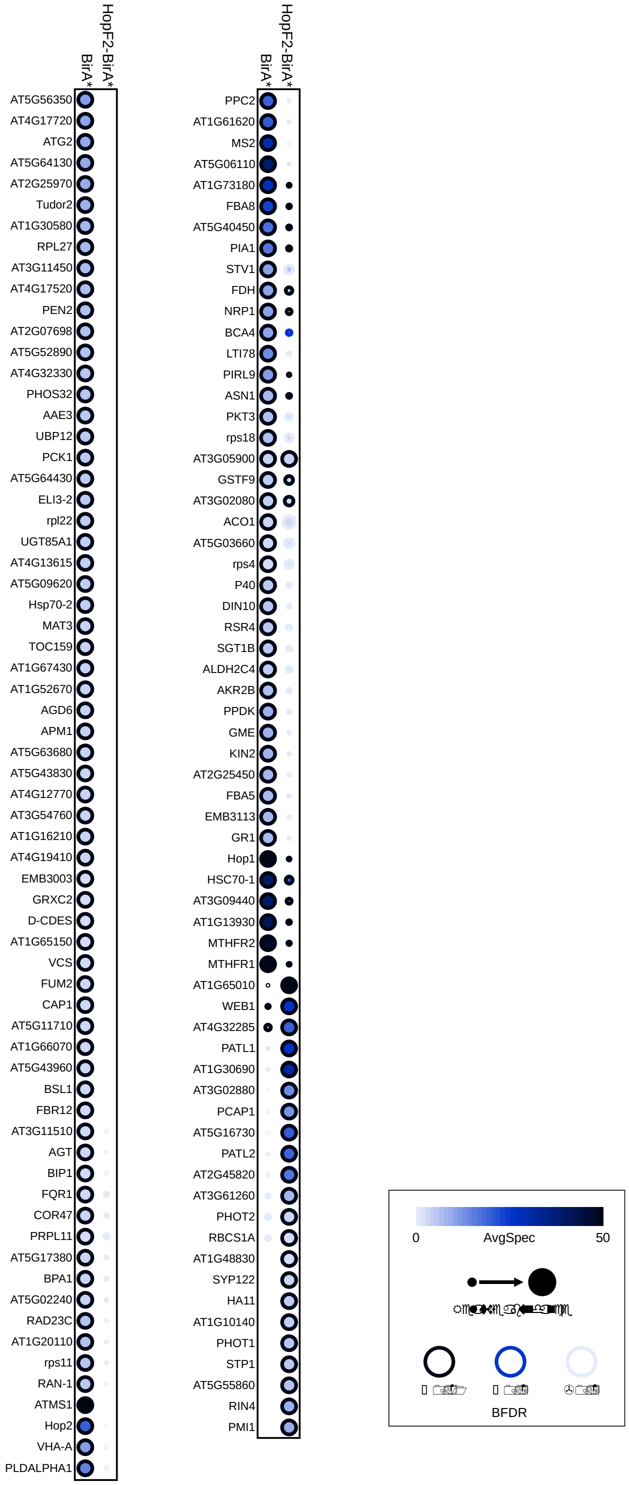

Supplementary Figure 2: Dot plot showing total list of prey proteins identified as significant interactors of BirA* and HopF2-BirA* by SAINT analysis.

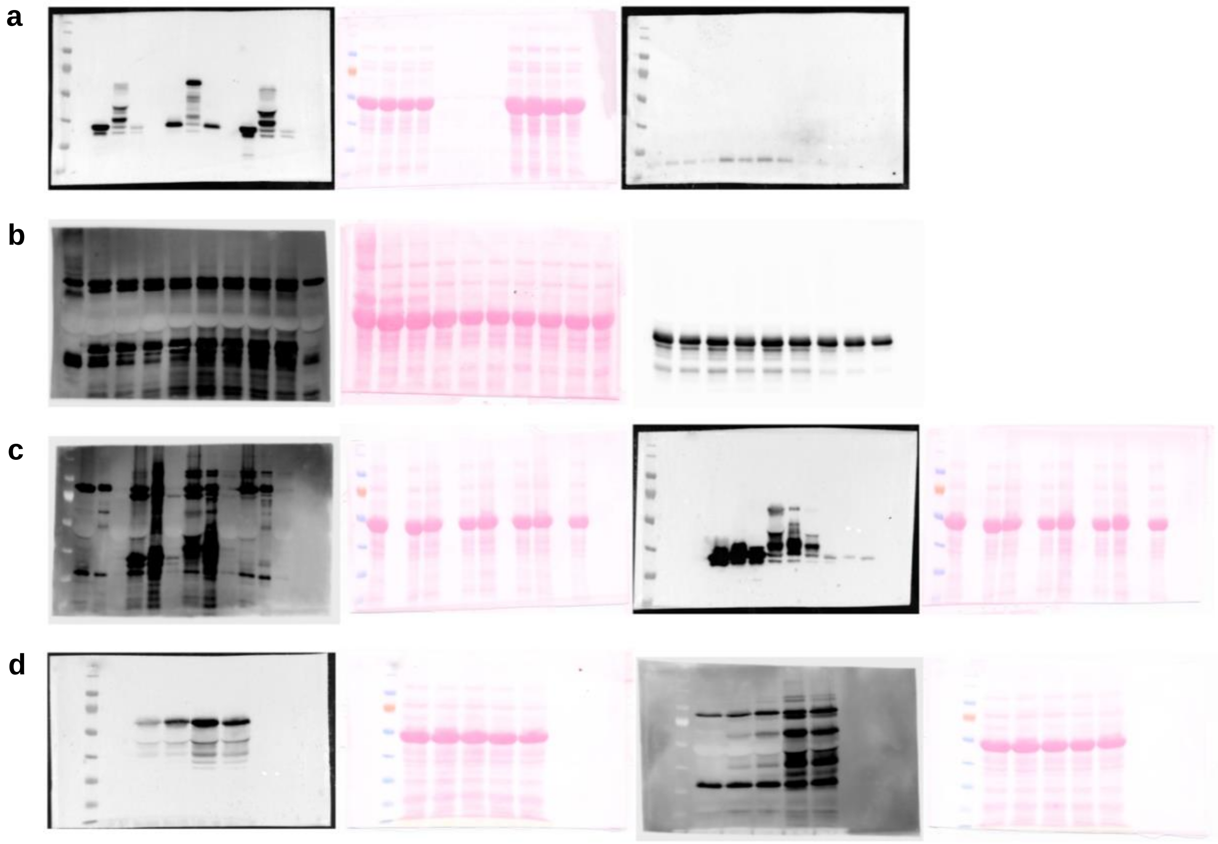

Supplementary Figure 3: Uncropped Images

(a) Images used in Figure 1a. **(b)** Images used in Figure 1b. **(c)** Images used in Figure 1c. **(d)** Images used in Supplementary Figure 1.

Supplementary Table 1: Significant Preys identified in BirA* but not in HopF2-BirA*

Preys	Common Name	Localization[a,b]	Identified by AP-MS[c,d]
At5g56350		Non-membrane	
At4g17720		Non-membrane	
At3g51800	ATG2	Non-membrane	
At5g64130		Membrane	
At2g25970		Non-membrane	
At5g61780	Tudor2	Non-membrane	
At1g30580		Non-membrane	
At5g40950	RPL27	Non-membrane	
At3g11450		N/A	
At4g17520	RGGB	Non-membrane	
At2g07698		Membrane	
At5g52890		N/A	
At2g44490	PEN2	Membrane	
At5g54430	PHOS32	Membrane	
At4g32330	WDL5	Non-membrane	
At3g48990	AAE3	Non-membrane	
At5g64430		Non-membrane	
At4g37990	ELI3-2	N/A	
At4g37870	PCK1	Membrane	
At5g06600	UBP12	Non-membrane	
At5g02490	Hsp70-2	Membrane	Yes (gi|12323026)
At4g13615		N/A	
At5g09620		N/A	
At1g22400	UGT85A1	Non-membrane	
AtCg00810	rpl22	Non-membrane	
At2g36880	MAT3	Membrane	
At4g02510	TOC159	Membrane	
At1g67430	RPL17B	Membrane	
At1g52670	BLP1	Non-membrane	
At3g53710	AGD6	Non-membrane	
At4g33090	APM1	Membrane	
At4g19410	PAE7	Membrane	
At4g12770		Membrane	
At3g54760		Non-membrane	
At1g16210		N/A	
At5g43830		Non-membrane	
At5g63680		Membrane	
At5g40370	GRXC2	Non-membrane	
At1g34430	EMB3003	Membrane	
At1g48420	D-CDES	Non-membrane	
At1g65150		N/A	
At3g13300	VCS	Non-membrane	Yes (gi|9294536)
At4g34490	CAP1	Non-membrane	
At4g03080	BSL1	Membrane	
At5g43960		Non-membrane	
At5g11710		Non-membrane	
At1g66070	eIF3j	Membrane	
At5g50950	FUM2	Non-membrane	

At1g26630	FBR12	Non-membrane	
At3g11510	RPS14B	Non-membrane	
At2g13360	AGT	Membrane	
At5g28540	BIP1	Membrane	
At5g54500	FQR1	Membrane	Yes (gi|8885593)
At1g20440	COR47	Non-membrane	Yes (gi|556474)
At1g32990	PRPL11	Membrane	
At5g17380	HACL	Non-membrane	
At5g16840	BPA1	Membrane	
At5g02240		Membrane	
At5g20010	RAN-1	Membrane	
AtCg00750	rps11	Non-membrane	
At3g02540	RAD23C	Non-membrane	
At1g20110	FYVE1	Membrane	
At3g24170	GR1	Non-membrane	
At2g33800	EMB3113	Membrane	
At4g26530	FBA5	Non-membrane	
At2g25450	GSL-OH	Non-membrane	
At5g15970	KIN2	Membrane	
At5g28840	GME	Non-membrane	
At4g15530	PPDK	Non-membrane	
At2g17390	AKR2B	Membrane	
At3g24503	ALDH2C4	Non-membrane	
At4g11260	SGT1B	Non-membrane	
At5g01410	RSR4	Membrane	
At5g20250	DIN10	N/A	
At1g72370	P40	Membrane	
AtCg00380	rps4	Non-membrane	
At5g03660		Membrane	
At4g35830	ACO1	Membrane	
AtCg00650	rps18	Non-membrane	
At2g33150	PKT3	Membrane	
At5g52310	LT178	Non-membrane	Yes (gi|16390)
At3g53020	STV1	Non-membrane	
At5g06110	ATGLSA2	N/A	
At5g17920	ATMS1	Membrane	
At1g62740	Hop2	Membrane	
At1g78900	VHA-A	Membrane	Yes (gi|2266990)
At3g15730	PLDALPHA1	Membrane	
At3g03780	MS2	Membrane	
At2g42600	PPC2	Membrane	
At1g61620	CSU1	Non-membrane	
At1g70410	BCA4	Membrane	

[a] Membrane or non-membrane based on subcellular localization information on Uniprot.org.
[b] 43.33% of BirA*-specific preys are membrane localized
[c] Identified in the AP-MS data of Hurley et al., 2014, in at least one HopF2 replicate and not in the negative control[15]
[d] 6.59% of BirA*-specific preys were also identified by AP-MS[15].

Supplementary Table 2: Significant Prey proteins common to HopF2-BirA* and BirA*

Preys	Common Name	Localization[a,b]	Identified by AP-MS[c,d]
At1g65010▼		Non-membrane	
At2g26570▼	WEB1	Membrane	
At4g32285▼		Membrane	Yes (gi|2864615)
At3g05900∆		Membrane	
At3g02080¥	RPS19A	Non-membrane	
At2g30860¥	GSTF9	Membrane	
At3g47340¥	ASN1	N/A	
At3g11330¥	PIRL9	N/A	
At1g12270¥	Hop1	Non-membrane	
At2g03440¥	NRP1	N/A	
At5g14780¥	FDH	Non-membrane	
At2g20630¥	PIA1	N/A	
At5g40450¥		N/A	
At3g52930¥	FBA8	Membrane	
At1g73180¥		Membrane	
At5g02500¥	HSC70-1	Membrane	
At3g09440¥	HSC70-3	Membrane	Yes (gi|3043415)
At1g13930¥		Membrane	
At2g44160¥	MTHFR2	Non-membrane	
At3g59970¥	MTHFR1	Non-membrane	

[a] Membrane or non-membrane based on subcellular localization information on Uniprot.org.
[b] 45% of preys common to HopF2-BirA* and BirA* are membrane localized
[c] Identified in the AP-MS data of Hurley et al., 2014, in at least one HopF2 replicate and not in the negative control[15]
[d] 10% of preys common to HopF2-BirA* and BirA* were also identified by AP-MS[15].
▼ Higher abundance in HopF2-BirA*
∆ Equal abundance in HopF2-BirA* and BirA*
¥ Higher abundance in BirA*
